# Primary Chest Wall Abscess Caused by Pseudomonas aeruginosa and Staphylococcus epidermidis

**DOI:** 10.7759/cureus.48544

**Published:** 2023-11-08

**Authors:** Rui Soares Correia, José Pedro Fonseca, Marlene Delgado

**Affiliations:** 1 Department of Internal Medicine, Centro Hospitalar Tondela - Viseu, Viseu, PRT

**Keywords:** staphylococcus epidermidis, pseudomonas aeruginosa, costochondritis, chest wall, thoracic abscess

## Abstract

Primary abscess of the thoracic wall is a very rare condition that occurs spontaneously due to hematogenous dissemination of bacterial, fungal, or mycobacterial pathogens, of which *Mycobacterium tuberculosis* is the most frequent agent. The authors describe a rare case of primary abscess of the thoracic wall. The patient presented with a painful, growing mass in the chest wall that later fistulized to the skin, draining a purulent exudate. Extensive analytical and imagiological workup was performed, showing no changes other than an expansive soft tissue formation extending from the skin surface and destructing the cartilage of the 7th right costal arch. Culture of the purulent exdudate identified *Pseudomonas aeruginosa* and *Staphylococcus epidermidis*. The patient improved under directed antibiotic treatment. The diagnosis of a primary abscess of the thoracic wall and the causative agents contribute to the rarity of this case.

## Introduction

An abscess of the thoracic wall can arise because of hematogenous dissemination of pathogenic microorganisms (primary infection), or it can occur either as a complication of thoracic open trauma or surgery or as a result of contiguous pulmonary or pleural infection (secondary infection). In contrast to secondary infection, primary abscess of the thoracic wall is very rare, and hematogenous dissemination of mycobacteria is its most prevalent cause [1]. Primary abscess of the thoracic wall with non-tuberculous etiology is an even rarer clinical condition, with only a few sporadic cases reported in the literature. An abscess of the thoracic wall may involve only the soft tissues, but it can spread and cause osteomyelitis of the rib(s) or costochondritis with consequent bone destruction [2]. Treatment varies according to the extent of the infection and includes prolonged antibiotic therapy, which may need to be combined with surgical procedures. The causative agents, *Pseudomonas aeruginosa* and *Staphylococcus epidermidis*, contribute to the rarity of this case as no other cases of a primary abscess of the thoracic wall due to these agents were found in the literature. This article was previously presented as a poster at the 27th National Congress of Internal Medicine in October 2021.

## Case presentation

A 73-year-old man with a history of type 2 diabetes mellitus (hemoglobin (Hb)A1C of 7.6%) and hypertension, treated with vildagliptin/metformin and olmesartan/amlodipine was referred to the medical consultation with a history of a growing mass in the anteroinferior portion of the chest wall during the last two months that later fistulized to the skin, draining a purulent exudate. The mass was painful, with a stable size after taking amoxicillin/clavulanic acid for seven days. The patient denied fever, chills, chest trauma, surgical or dental procedures, implantation of medical devices, or recent infections or hospitalizations. However, he had undergone a polypectomy three weeks before the onset of symptoms, and the histology of the excised colon polyps revealed adenomas with low- and high-grade dysplasia. On physical examination, the patient was afebrile, and a firm mass in the anteroinferior portion of the right chest wall with mild signs of inflammation and drainage of purulent exudate was noted. Further evaluation included blood tests, with negative inflammation biomarkers and serologies (Table 1). 

**Table 1:** Summary of laboratory results CRP: C-Reactive Protein; ADA: adenosine deaminase; HIV: human immunodeficiency virus; CMV: cytomegalovirus; EBV: Epstein Barr virus; PSA: Prostate-specific antigen

Test		Values	Reference range
Hemoglobin (g/dL)		12.0	14.0-18.0
Leukocytes (/μL)		6900	4500-11500
Erythrocyte sedimentation rate (mm)		6.0	0.0-10.0
CRP (mg/dL)		0.4	0.0-0.5
Serum protein electrophoresis		Normal	
ADA (UI/L)		26.0	4.8-23.1
Interferon Gamma (Quantiferon TB-Gold)		Negative	
Serologies	Brucella abortus	Negative	
	HIV	Negative	
	Hepatitis B virus	Negative	
	Hepatitis C virus	Negative	
	Treponema pallidum	Negative	
	CMV	Negative	
	EBV	Negative	
PSA (ng/mL)		0.6	0.0-4.0

**Figure 1:**
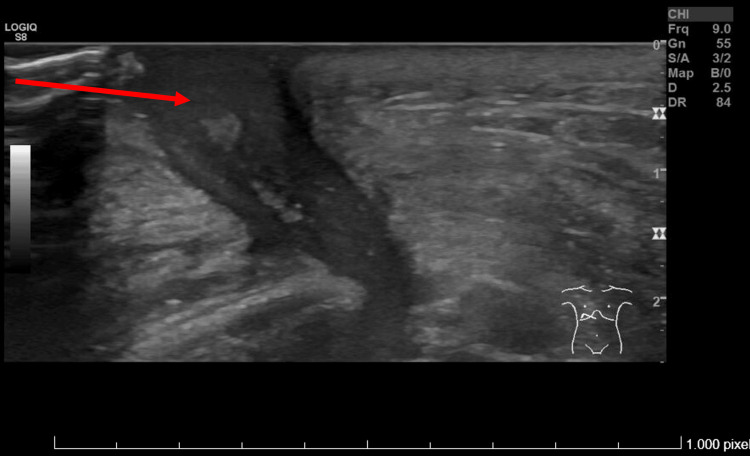
Chest ultrasound image The red arrow shows the chest wall mass.

Ultrasound (Figure 1) revealed a hypoechoic elongated mass with poorly defined borders with central vascularization that extended from the skin to the costal cartilage, which was eroded.

**Figure 2:**
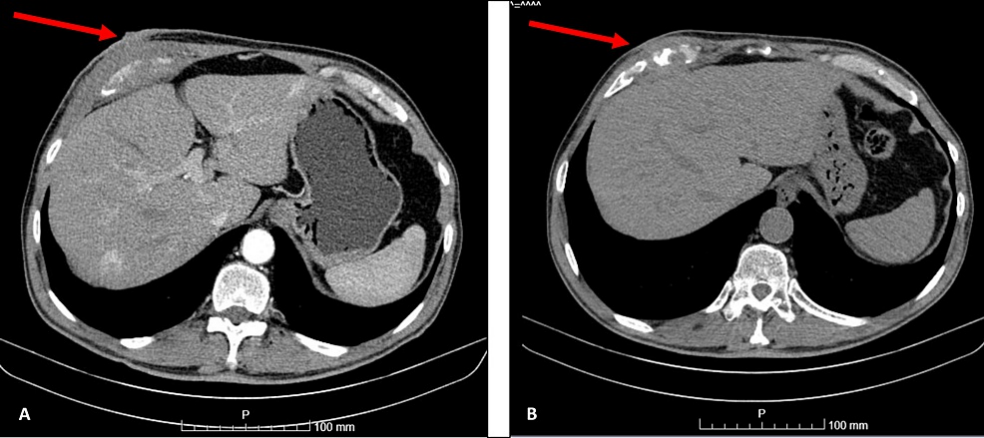
CT scan A: With intravenous contrast; B: without intravenous contrast. Red arrows show the chest wall mass.

Thoraco-abdominopelvic computed tomography (CT) (Figure 2) showed an expansive soft tissue formation measuring 89 × 46 × 70 mm with poorly defined borders, extending from the skin surface and destructing the cartilage of the 7th right costal arch with thickening of the adjacent rib muscles. 

Histological examination of the mass biopsy revealed fibrous connective tissue with a polymorphous inflammatory infiltrate, compatible with a fistulous tract, and no neoplastic structures were identified. Culture of the lesion exudate identified the growth of *Pseudomonas aeruginosa* and *Staphylococcus epidermidis*, with a negative culture for mycobacteria. Antibiotic therapy was initiated according to the antibiogram with amoxicillin + clavulanic acid and levofloxacin for 21 days, resulting in progressive regression of the mass and suppuration. Two months after completing therapy, the patient remained asymptomatic, and a chest CT scan showed only slight soft tissue densification at the level of the anterior 7th costal arch, while the bone scan showed no abnormalities.

## Discussion

Primary abscess of the chest wall is a rare condition that requires the exclusion of secondary causes. Early diagnosis is essential, as it can progress to serious infections, especially in older or immunocompromised patients. Adequate antibiotic therapy and surgical treatment affect the prognosis [3]; however, the indication for surgery depends on the extent of the infection and the response to antibiotic therapy.

Primary abscesses of the chest wall are generally caused by *Mycobacterium tuberculosis*, while cases of abscesses caused by other bacterial agents have been reported very sporadically in the literature. The agents identified in these cases include *Staphylococcus aureus, Streptococcus, Escherichia coli, Salmonella sp., Bartonella henselae, Actinomyces, and Bacteroides.* No described cases of abscesses caused by *Pseudomonas aeruginosa* or *Staphylococcus epidermidis* were found.

*Pseudomonas aeruginosa* is an opportunistic pathogen infecting immunocompromised individuals, such as patients with cystic fibrosis, bronchiectasis, neutropenia, burns, neoplasms, acquired immunodeficiency syndrome, organ transplantation, and uncontrolled diabetes mellitus and those admitted to intensive care units [4]. In addition, it is commonly found in the environment, especially in freshwater, and can cause community-acquired infections [5].

*Staphylococcus epidermidis* is typically present as normal skin flora. However, its pathogenicity has been demonstrated, and it is often implicated in surgical implant infections, endocarditis, and skin and soft tissue infections. It is currently recognized as the most frequent cause of nosocomial sepsis. Increasing evidence suggests that it should not always be considered a contaminant in cases of skin and soft tissue infections. Therefore, if a coagulase-negative *Staphylococcus* is isolated in a bacterial culture from a suspected infectious skin or soft tissue lesion, the organism should be considered a pathogenic agent for the infection, and targeted antibiotic therapy should be instituted. Predisposing factors for skin and soft tissue infections by coagulase-negative *Staphylococcus* include advanced age, immunocompromised status, particularly in patients with diabetes mellitus [6], and surgical implants or medical devices, which serve as a nidus for infection [7].

In this presented clinical case, it was not possible to identify the primary source of infection. However, given the temporal correlation between the polypectomy and the onset of the chest abscess, it is possible that this procedure may have been responsible for transient bacteremia with subsequent focalization in the chest wall. Despite gastrointestinal infections by *Pseudomonas aeruginosa* being uncommon, they have been described as post-endoscopic retrograde cholangiopancreatography (ERCP) cholangitis, enteritis, and enterocolitis. Except for diabetes mellitus, which increased this patient’s susceptibility to infections [8], there were no other clinical conditions predisposing the patient to infection by these microbiological agents.

Considering the isolation of *Pseudomonas aeruginosa*, it is not possible to conclude that *Staphylococcus epidermidis* was only a contaminant, so it was considered a pathogen and treated as such.

## Conclusions

This case report describes a rare case of primary chest wall abscess of bacterial origin with the aim of reminding the medical community to consider this pathological entity in the differential diagnosis of chest wall masses. Although this is a rare clinical condition, early diagnosis, identification of causative agents, and adequate treatment are crucial, as progression to severe infections can occur, especially in older and immunocompromised patients. Unusual pathogens should always be considered, as adequate and timely antibiotic therapy, and eventually, surgical approaches affect the prognosis.
